# 

**Published:** 1878-09

**Authors:** 


### Table of Contents.

### ORIGINAL COMMUNICATIONS.

The Metric System in Medicine. *By Edw. Wegglelworth, A. M., M.D., Boston*.......... 321
The Principles of Medical Practice, Formerly and Now. *By J. Dickson Smith, M.D., Atlanta, Ga*.......... 329
On the Treatment of Blennorrhagic Affections by Gurjun Balsam. Translated from the French. *By R. J. Nunn, M.D., Paris France*.......... 335

### SELECTIONS.

Preventive Treatment of Yellow Fever in Havana.......... 341
A Case of Traumatic Tetanus Treated by Physostigma.......... 344
Dysmenorrhœa—its Treatment.......... 346
Case of Ovariotimy.......... 348
Perineorraphy.......... 349
The use of Common Warts of the hand in Skin Grafting.......... 354
Treatment of Accidental Hemorrhage.......... 355

### CORRESPONDENCE.

European Correspondence.......... 365, 369

### EDITORIAL.

The Metric System.......... 373

### EXCERPTA.

The Chinese Question in its Medical Bearing.......... 375
Seven Good Rules for Preserving the Eye-sight.......... 377
Official or Officinal?.......... 378
Travel in Phthisis.......... 380
Lead Poisoning.......... 381
The McDowell Monument.......... 381
Phimosis as a cause of Rupture in Children.......... 381
A Man who Burst.......... 382
Venereal Sores—Iodoform.......... 382
Wounds.......... 383
Treatment of Diphtheria by the French School.......... 383
The Periodicity of Epidemics.......... 384
The Periodicity of Yellow Fever.......... 384

